# Detectability and healthcare implications of generative AI–synthesized chest radiographs: a blinded radiologist reader study

**DOI:** 10.3389/fmed.2026.1901949

**Published:** 2026-07-15

**Authors:** Jinghang Wang, Ruixin Wang, Qijia Yi, Hongyong Tang, Li Fan, Shunan Lin, Wenjing He, Dan Peng, Jun-Jie Yang, Jun Liu

**Affiliations:** 1 Department of Radiology, The Second Affiliated Hospital of Xinjiang Medical University, Ürümqi, Xinjiang, China; 2 Department of Radiology, The Second Xiangya Hospital of Central South University, Changsha, Hunan, China; 3 Department of Radiology, The First Hospital of Lanzhou University, Lanzhou, Gansu, China; 4 Department of Radiology, Pengan County People’s Hospital, Nanchong, Sichuan, China

**Keywords:** chest radiography, gemini-3-pro-image-preview, generative artificial intelligence, gpt-image-2, healthcare research integrity, synthetic medical images

## Abstract

Generative artificial intelligence (GenAI) is increasingly explored for medical image synthesis, medical education, and dataset augmentation; however, the detectability and reader-perceived visual authenticity of synthetic chest radiographs generated by accessible multimodal models remain insufficiently understood. This study evaluated synthetic disease-specific chest radiographs generated by gpt-image-2 and gemini-3-pro-image-preview, hereafter referred to as the GPT-image model and the Gemini-image model, respectively, using two generation strategies: text-only generation and image-conditioned generation based on age- and sex-matched normal conditioning radiographs. We included 320 real disease-positive frontal chest radiographs covering cardiomegaly, pneumothorax, pleural effusion, and pneumonia, together with 320 matched normal conditioning radiographs. Synthetic and real disease-positive radiographs were randomized and independently assessed by four radiologists in a blinded single-image reader study, followed by paired evaluation of previously undetected image-conditioned synthetic radiographs. Image-level similarity was assessed using the structural similarity index measure (SSIM), learned perceptual image patch similarity (LPIPS), and Fréchet Inception Distance (FID). Image-conditioned generation had significantly lower AI detection rates than text-only generation (34.0% vs. 56.1%) and consistently lower FID values. Synthetic radiographs generated by the GPT-image model were less frequently detected than those generated by the Gemini-image model and showed higher image-level structural and perceptual similarity to the conditioning radiographs. Paired comparison improved detection of previously undetected image-conditioned synthetic radiographs. These findings indicate that image-conditioned GenAI can produce synthetic chest radiographs that may appear visually authentic to radiologists, highlighting the need for transparent labeling, provenance tracking, expert review, and controlled integration of synthetic medical images into healthcare, education, and research workflows.

## 1 Introduction

Generative artificial intelligence (GenAI) has increasing potential in medical imaging and healthcare. Synthetic medical images may support dataset augmentation, rare-disease supplementation, algorithm development, medical education, and simulation-based research (1–4). Conventional image synthesis methods, including generative adversarial networks and diffusion models, have shown promise in chest radiography and other imaging tasks (5–7). However, these approaches often require large domain-specific datasets, substantial computational resources, and specialized training pipelines. They are also often optimized for specific anatomic regions or narrow tasks and offer limited control over detailed lesion characteristics (8–10).

Large multimodal models with image-generation capabilities provide a more accessible route for medical image synthesis (11, 12). These models can generate images from natural-language prompts without requiring users to train dedicated generative models (10, 13). This accessibility enables users to specify imaging findings and lesion features in plain language. It may facilitate medical education, dataset construction, and exploratory healthcare AI research. Importantly, when prompts are derived from real radiology reports rather than manually designed generic descriptions, synthetic image generation may better reflect clinical reporting language and disease-specific imaging descriptions. Such report-informed synthesis may therefore provide a relevant framework for evaluating GenAI-generated medical images.

At the same time, the increasing visual realism and accessibility of generative models raise important concerns for clinical and research workflows. Synthetic radiographs that closely resemble real examinations may contaminate imaging datasets, be inappropriately used in education or research, or create uncertainty regarding image provenance if not transparently labeled (14–16). Previous influential studies have demonstrated the relevance of this issue. Bluethgen et al. showed that vision-language foundation models can generate radiographically plausible chest radiographs from free-text medical prompts (8). Tordjman et al. further reported that expert radiologists achieved only moderate performance in distinguishing large language model–generated radiographs from real radiographs (14). These findings underscore the need to systematically evaluate the visual realism, detectability, and provenance of synthetic medical images before their use in education, dataset construction, or research.

Most prior evaluations have focused on synthetic images generated from text prompts alone (13, 17). However, newer multimodal image-generation models can also use conditioning images as inputs (18–20). Image-conditioned generation may preserve patient-like anatomy, projection characteristics, and background radiographic texture more effectively than text-only generation (19). This preservation may make synthetic chest radiographs more difficult to identify. Among recently available general-purpose image-generation models, gpt-image-2 and gemini-3-pro-image-preview have attracted substantial attention because of their strong performance in broad multimodal and image-generation tasks. We refer to these models as the GPT-image and Gemini-image models, respectively. Nevertheless, despite their accessibility and rapid adoption in non-medical contexts, their performance in disease-specific synthetic chest radiograph generation remains insufficiently evaluated. In particular, it remains unclear how these models perform in disease-specific synthetic chest radiograph generation when prompted by real radiology-report findings and when matched normal radiographs are used as conditioning inputs.

Therefore, this study evaluated the detectability and reader-perceived visual authenticity of finding-guided synthetic chest radiographs generated by the GPT-image and Gemini-image models. Imaging findings extracted from real radiology reports were used to construct report-informed prompts, and two synthesis strategies were compared: text-only generation and image-conditioned generation based on age- and sex-matched normal conditioning radiographs. AI-generated and real disease-positive radiographs were randomized and assessed by radiologists in a blinded reader study, with quantitative image metrics used to complement visual evaluation. By evaluating two widely discussed general-purpose multimodal image-generation models in a chest radiography setting, this study provides evidence relevant to the transparent labeling, provenance tracking, and controlled use of synthetic medical images in education and research.

## 2 Materials and methods

This retrospective study was approved by the Institutional Review Board of our institution, which waived the requirement for patients’ informed consent referring to the Council for International Organizations of Medical Sciences (CIOMS) guidelines. All data were anonymized prior to analysis to ensure participant confidentiality.

### 2.1 Study overview and case selection

**Figure 1:**
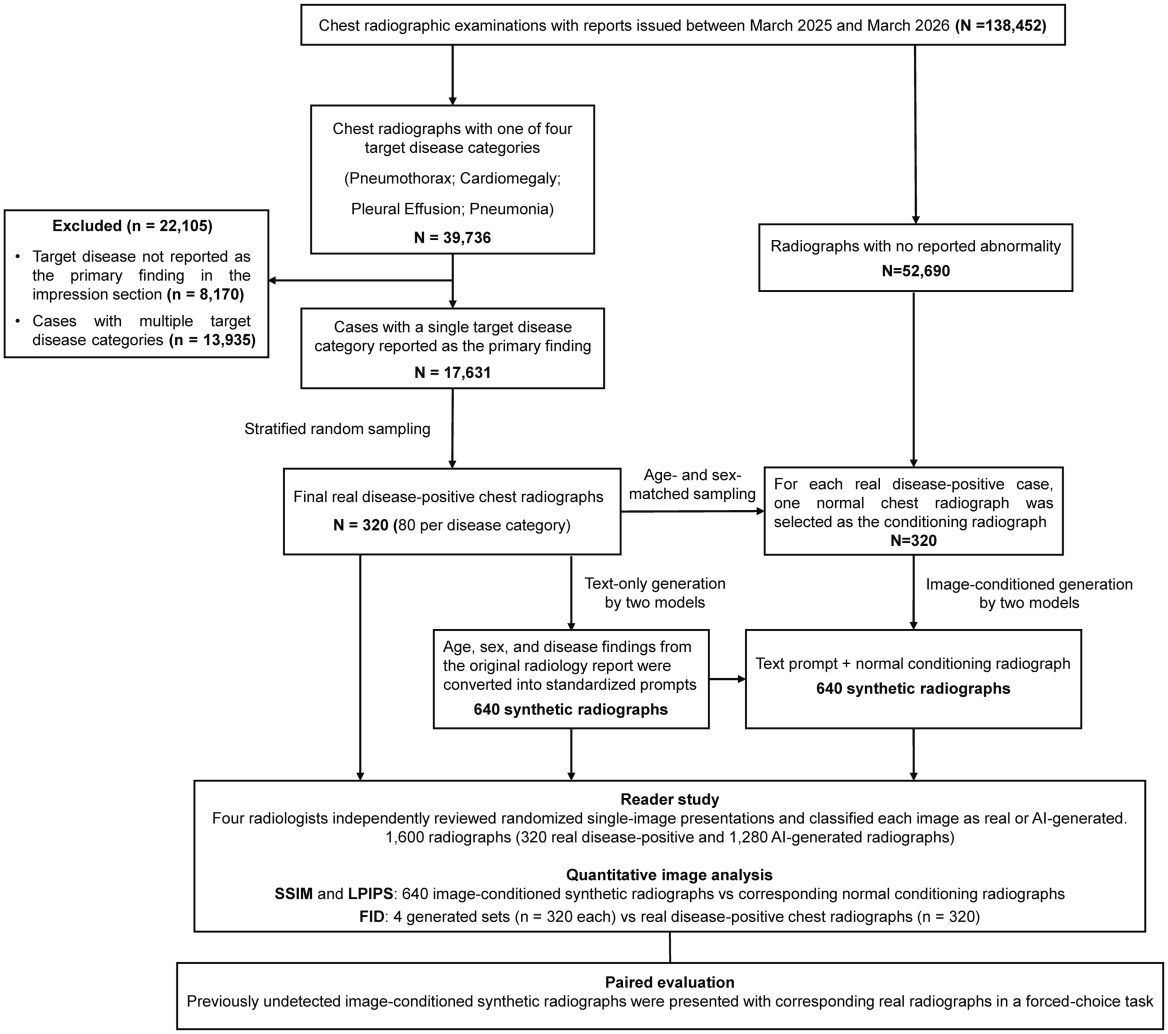
Overall study workflow and case selection. A total of 320 real disease-positive chest radiographs and 320 age- and sex-matched normal conditioning radiographs were included. Report-informed prompts were used to generate synthetic radiographs with the GPT-image and Gemini-image models using text-only and image-conditioned strategies, followed by blinded reader assessment, paired evaluation, and quantitative image analysis.

The overall study workflow and case selection process are shown in Figure 1. This was a single-center retrospective study of report-informed image generation and blinded provenance reader assessment, designed to compare the perceived authenticity and AI-origin detection rates of disease-specific synthetic chest radiographs generated using two synthesis strategies. All source radiographs were retrieved from the radiology PACS of our institution and consisted of previously acquired frontal chest radiographs. Before export, all images were fully anonymized by removing patient identifiers, including name, medical record number, and date of birth, and were exported as high-quality JPG files.

We included 320 real disease-positive chest radiographs with imaging findings documented in the original radiology reports. For image-conditioned generation, we additionally selected 320 normal conditioning radiographs with no reported abnormalities, matched to the source disease-positive cases by age and sex. Age matching was performed within the same decade, and all patients were aged ≥20 years. The four disease categories were cardiomegaly, pneumothorax, pleural effusion, and pneumonia.

### 2.2 Chest radiograph synthesis using general-purpose multimodal generative AI models

Image synthesis was performed using the GPT-image and Gemini-image models. All model calls were made through the application programming interfaces (APIs) rather than manual web-based prompting. For each disease-positive case, the patient’s age, sex, and imaging findings extracted from the original radiology report were converted into a standardized text prompt instructing the model to generate a frontal chest radiograph.

Two synthesis strategies were evaluated. In synthesis mode 1 (text-only generation), the model generated a diseased chest radiograph using text information only. In synthesis mode 2 (image-conditioned generation), the same text prompt was combined with the corresponding age- and sex-matched normal frontal chest radiograph, and the model was instructed to generate a disease appearance consistent with the text description. All source radiographs were center-cropped to preserve the short side and converted to a 1:1 aspect ratio before use. All synthetic radiographs were generated as PNG files with a 1:1 aspect ratio. Detailed image preprocessing steps are summarized in Supplementary Appendix S1. The standardized prompts and API calling parameters for the GPT-image and Gemini-image models are provided in Supplementary Tables S1, S2, respectively.

### 2.3 Reader study for synthetic radiograph detection

Four board-certified radiologists, with 20, 16, 13, and 4 years of experience for readers 1–4, respectively, participated in the single-image reader assessment to judge whether each radiograph was AI-generated or real. The initial reader study included both real disease-positive radiographs and synthetic disease-positive radiographs. Specifically, the 320 real disease-positive chest radiographs and all synthetic radiographs generated by the two models and two synthesis strategies were pooled, randomized, and presented one at a time. Readers were blinded to image provenance and were not informed of the number or proportion of real and synthetic images, nor of the model or synthesis mode used for synthetic images. Age, sex, disease category, and disease-specific radiological findings were provided, and readers were asked to judge whether each image was synthetic or real. Each image was accompanied by one disease-specific radiological finding most directly relevant to the assigned disease category. When available, this finding included quantitative or semi-quantitative descriptors from the original radiology report, such as cardiothoracic ratio for cardiomegaly or estimated lung compression percentage for pneumothorax. A binary response was recorded for each image.

After the initial single-image assessment, image-conditioned synthetic radiographs that had not been identified as AI-generated by each reader were included in a paired forced-choice evaluation. Each previously undetected reader–image instance was paired with the corresponding real disease-positive radiograph and shown side by side to the same reader, who was asked to identify the AI-generated radiograph.

### 2.4 Quantitative image analysis

Quantitative image metrics were calculated using Python-based image analysis packages to complement the reader study. For image-conditioned generation, the structural similarity index measure (SSIM) and learned perceptual image patch similarity (LPIPS) were computed between each generated image and its corresponding matched normal conditioning radiograph used as the conditioning input, separately for the GPT-image and Gemini-image models. These metrics were used to assess structural and perceptual similarity to the conditioning radiograph rather than diagnostic correctness or clinical disease severity. SSIM was calculated using scikit-image, and LPIPS was calculated using the Python lpips package with PyTorch. Fréchet Inception Distance (FID) was calculated between each synthetic disease-positive radiograph set and the corresponding real disease-positive radiograph set for each model and synthesis strategy using torchmetrics with PyTorch. Higher SSIM and lower LPIPS indicate greater similarity to the conditioning radiograph, whereas lower FID indicates closer distributional similarity to real disease-positive radiographs. Details of the FID pipeline, including the Inception feature extractor, RGB handling, and image resolution, are provided in Supplementary Appendix S2.

### 2.5 Statistical analysis

Statistical analyses were performed using Python v3.10 (SciPy v1.5.4 and statsmodels v0.13.5) and R version 4.5.2 with the lme4 and emmeans packages. Continuous variables are presented as mean ± standard deviation, and categorical variables as number (percentage). Among synthetic radiographs, a generalized linear mixed model (GLMM) with a binomial distribution and logit link was used to assess the association of synthesis mode, generation model, disease category, age, and sex with AI detection rate, with random intercepts for reader and source disease-positive case. Each source disease-positive case corresponded to one real disease-positive radiograph. Odds ratios (ORs) and 95% confidence intervals (CIs) were calculated. Pairwise comparisons between groups were performed using estimated marginal means. Inter-reader agreement for the single-image reader study was assessed using Fleiss’ kappa across the four radiologists, and pairwise Cohen’s kappa with 95% confidence intervals was calculated for each reader pair.

Within the reader study, paired comparisons between text-only and image-conditioned generation were analyzed using exact McNemar tests. Holm correction was applied across all corresponding pairwise comparisons, including overall and disease-stratified comparisons for both models. For the paired evaluation, accuracy was defined as the proportion of trials in which the reader correctly identified the AI-generated radiograph; Wilson 95% CIs were calculated, and between-model differences were compared using the two-proportion z test. SSIM and LPIPS were compared between models using the Mann–Whitney U test. FID was calculated between the real disease-positive radiograph set and images generated under each synthesis strategy. To provide a reference baseline for FID, split-half FID was additionally calculated between two randomly partitioned halves of the real disease-positive radiograph set (160 vs. 160 images), and a 95% CI was estimated using 1,000 bootstrap resamples. All tests were two-sided, and *p* < 0.05 was considered statistically significant. To accompany significance testing, effect sizes were reported where applicable as adjusted odds ratios for GLMM analyses, absolute differences in proportions for binary outcomes, and mean between-model differences for continuous image-similarity metrics.

## 3 Results

### 3.1 Demographic characteristics

A total of 640 real source radiographs were included, comprising 320 real disease-positive radiographs and 320 matched radiographically normal conditioning radiographs. Overall, the mean age was 58.5 ± 14.2 years, and 45.6% of cases (292 of 640) were from female patients (Table 1).

**Table 1:** Demographic characteristics of real disease-positive radiographs and matched normal conditioning radiographs by disease category.

	Disease-positive radiographs		Matched normal conditioning radiographs		Total	
Disease category	Age (y)	No. of women	Age (y)	No. of women	Age (y)	No. of women
Total	58.6 ± 14.2	146/320 (45.6)	58.3 ± 14.1	146/320 (45.6)	58.5 ± 14.2	292/640 (45.6)
Cardiomegaly	58.8 ± 14.4	49/80 (61.3)	58.8 ± 14.5	49/80 (61.3)	58.8 ± 14.4	98/160 (61.3)
Pneumothorax	56.1 ± 13.4	27/80 (33.8)	56.2 ± 13.2	27/80 (33.8)	56.1 ± 13.3	54/160 (33.8)
Pleural effusion	58.7 ± 13.2	38/80 (47.5)	58.7 ± 13.2	38/80 (47.5)	58.7 ± 13.2	76/160 (47.5)
Pneumonia	60.6 ± 15.7	32/80 (40.0)	59.8 ± 15.3	32/80 (40.0)	60.2 ± 15.4	64/160 (40.0)

Age is shown as mean ± standard deviation; sex is shown as n/N (%).

### 3.2 AI detection rates and inter-reader agreement in the reader study

**Figure 2:**
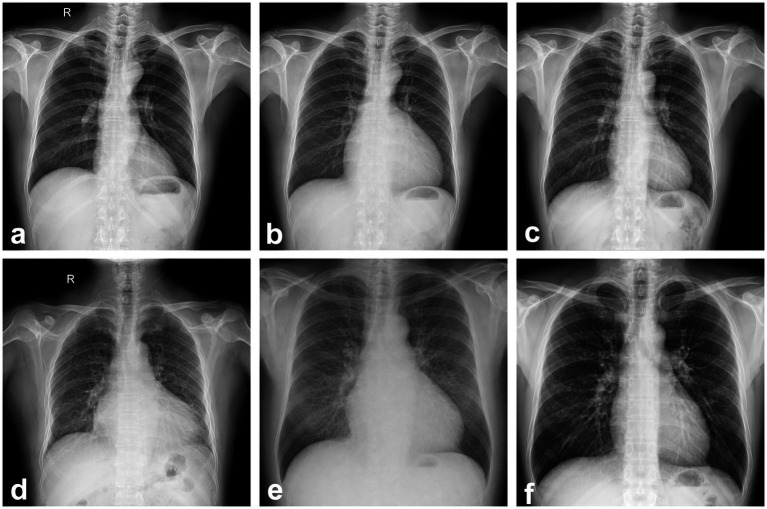
Representative cardiomegaly case and matched normal conditioning radiograph. **(a)** Normal conditioning radiograph from a 55-year-old man used for image-conditioned generation. **(b,c)** Image-conditioned synthetic radiographs generated by the GPT-image and Gemini-image models, respectively. **(d)** Real disease-positive radiograph from a 53-year-old man with cardiomegaly, with a cardiothoracic ratio of 0.65. **(e,f)** Text-only synthetic radiographs generated by the GPT-image and Gemini-image models, respectively.

Representative examples of the normal conditioning radiograph, synthetic disease-positive radiographs generated by the two models using image-conditioned and text-only strategies, and the corresponding real disease-positive radiograph are shown in Figure 2.

Using synthetic provenance as the positive class, the overall sensitivity for identifying synthetic radiographs was 45.0% (2,306/5120), whereas the specificity for identifying real disease-positive radiographs as real was 92.4% (1,183/1280), corresponding to a balanced accuracy of 68.7%.

**Figure 3:**
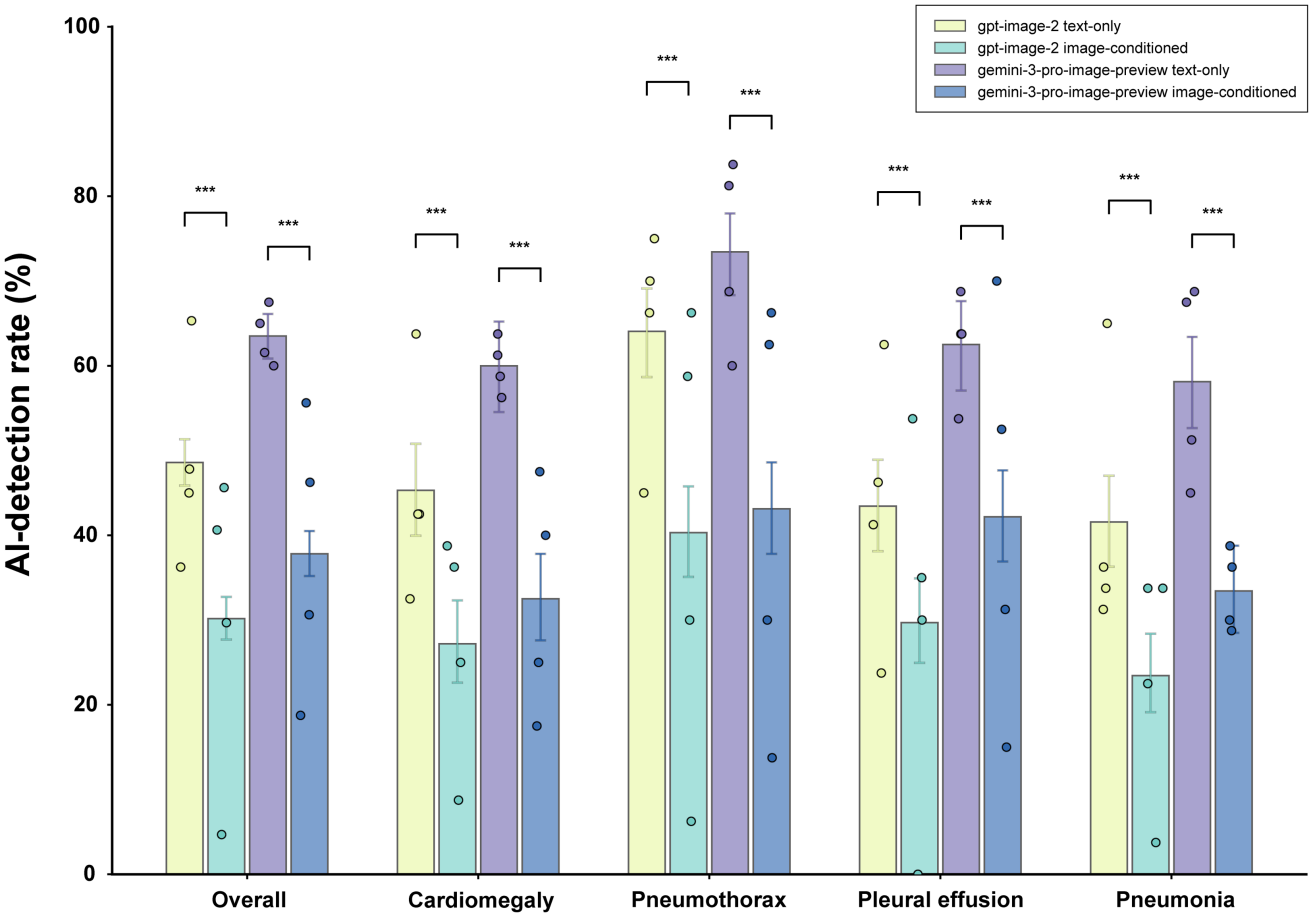
AI-detection rates across disease categories and generation methods. Bars show pooled AI-detection rates across the four radiologists, with 95% Wilson confidence intervals. Dots indicate individual radiologist performance. Brackets indicate pairwise comparisons; *** indicates adjusted *p* < 0.001 (McNemar test with Holm correction).

Among real disease-positive radiographs, the overall false-positive rate, defined as real disease-positive radiographs incorrectly judged as synthetic, was 7.6% (97/1280), as detailed in Supplementary Table S3. As shown in Figure 3, the overall AI detection rate was consistently higher for text-only than for image-conditioned generation for both models. For the GPT-image model, the overall AI detection rate was 48.7% (623/1280) for text-only generation and 30.2% (386/1280) for image-conditioned generation, corresponding to an absolute reduction of 18.5% with image-conditioned generation (McNemar test with Holm correction, adjusted *p* < 0.001). For the Gemini-image model, the corresponding rates were 63.5% (813/1280) and 37.8% (484/1280), respectively, corresponding to an absolute reduction of 25.7% (adjusted p < 0.001). This pattern was consistently observed across all four disease categories. Among disease categories, pneumothorax showed the highest AI detection rates, with rates of 64.1% (205/320) for text-only generation with the GPT-image model, 40.3% (129/320) for image-conditioned generation with the GPT-image model, 73.4% (235/320) for text-only generation with the Gemini-image model, and 43.1% (138/320) for image-conditioned generation with the Gemini-image model.

Inter-reader agreement was assessed separately within each synthesis method using Fleiss’ kappa and pairwise Cohen’s kappa. Agreement among interpretations under text-only generation was fair (Fleiss’ kappa = 0.311, 95% CI 0.275–0.345; pairwise Cohen’s kappa range, 0.214–0.461), whereas agreement under image-conditioned generation was slight to fair and overall lower (Fleiss’ kappa = 0.129, 95% CI 0.098–0.162; pairwise Cohen’s kappa range, −0.018 to 0.372). Together with the lower AI-detection rates, these findings support that image-conditioned generation radiographs were more difficult for radiologists to consistently recognize as AI-generated.

### 3.3 Generalized linear mixed-model analysis of factors associated with AI detection

GLMM analysis showed that image-conditioned generation was associated with significantly lower odds of being identified as synthetic than text-only generation (pooled unadjusted AI detection rate: 34.0% vs. 56.1%; absolute reduction, 22.1%; adjusted OR: 0.26; 95% CI: 0.23–0.30; *p* < 0.001; Table 2). The absolute reductions in AI detection rate for image-conditioned generation, overall and across model and disease subgroups, are shown in Supplementary Figure S1. Synthetic radiographs generated by the Gemini-image model were more likely to be identified as synthetic than those generated by the GPT-image model (pooled unadjusted AI detection rate: 50.7% vs. 39.4%; absolute difference, 11.3%; adjusted OR: 2.26; 95% CI: 1.59–3.20; *p* < 0.001). Among disease categories, pooled unadjusted AI detection rates were 55.2% for pneumothorax, 44.5% for pleural effusion, 41.3% for cardiomegaly, and 39.1% for pneumonia. In the adjusted GLMM, only pneumothorax was associated with higher odds of being identified as synthetic relative to cardiomegaly (adjusted OR: 3.25; 95% CI: 2.13–4.95; *p* < 0.001), whereas pleural effusion and pneumonia were not significantly different. Male sex was associated with lower odds of being identified as synthetic than female sex (adjusted OR: 0.75; 95% CI: 0.62–0.92; *p* = 0.006), while age group was not significant (*p* = 0.242).

**Table 2:** AI detection rates and GLMM results among synthetic chest radiographs.

Analysis	Contrast	AI detection rate	OR	95% CI	*p*-value
Overall effects					
Generation mode	Image-conditioned vs. Text-only	34.0% (870/2560) vs. 56.1% (1,436/2560)	0.26	0.23–0.30	< 0.001
Model	Gemini vs. GPT	50.7% (1,297/2560) vs. 39.4% (1,009/2560)	2.26	1.59–3.20	< 0.001
Disease category	Pleural effusion vs. Cardiomegaly	44.5% (569/1280) vs. 41.3% (529/1280)	0.91	0.61–1.37	0.652
	Pneumonia vs. Cardiomegaly	39.1% (501/1280) vs. 41.3% (529/1280)	0.84	0.56–1.27	0.414
	Pneumothorax vs. Cardiomegaly	55.2% (707/1280) vs. 41.3% (529/1280)	3.25	2.13–4.95	< 0.001
Age	>55 years vs. ≤ 55 years	Not applicable	1.14	0.92–1.41	0.242
Sex	Male vs. Female	Not applicable	0.75	0.62–0.92	0.006
Generation mode within model					
GPT-image model	Image-conditioned vs. Text-only	30.2% (386/1280) vs. 48.7% (623/1280)	0.32	0.27–0.39	< 0.001
Gemini-image model	Image-conditioned vs. Text-only	37.8% (484/1280) vs. 63.5% (813/1280)	0.22	0.18–0.26	< 0.001
Model within generation mode					
Text-only	Gemini vs. GPT	63.5% (813/1280) vs. 48.7% (623/1280)	2.40	2.00–2.88	< 0.001
Image-conditioned	Gemini vs. GPT	37.8% (484/1280) vs. 30.2% (386/1280)	1.63	1.35–1.97	< 0.001

The GLMM used a binomial logit model with random intercepts for reader and source case. Reference categories were text-only generation, GPT-image model, female sex, age ≤55 years, and cardiomegaly. AI detection rates are pooled unadjusted rates and are shown as % (n/N). ORs were estimated from the adjusted GLMM and may differ from crude comparisons of pooled rates; disease-category ORs are conditional estimates from the interaction model. OR, odds ratio; CI, confidence interval; GLMM, generalized linear mixed model.

In model-stratified comparisons, the lower detectability of image-conditioned generation remained significant for both the GPT-image model (pooled unadjusted AI detection rate: 30.2% vs. 48.7%; adjusted OR: 0.32; 95% CI: 0.27–0.39; *p* < 0.001) and the Gemini-image model (37.8% vs. 63.5%; adjusted OR: 0.22; 95% CI: 0.18–0.26; *p* < 0.001). Likewise, synthetic radiographs generated by the Gemini-image model remained more likely to be identified as synthetic than those generated by the GPT-image model under both text-only generation (63.5% vs. 48.7%; adjusted OR: 2.40; 95% CI: 2.00–2.88; *p* < 0.001) and image-conditioned generation (37.8% vs. 30.2%; adjusted OR: 1.63; 95% CI: 1.35–1.97; *p* < 0.001).

### 3.4 Quantitative image-level analysis

**Figure 4:**
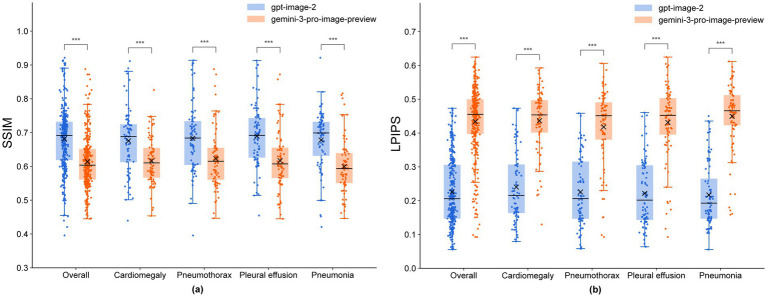
Similarity metrics for image-conditioned synthetic chest radiographs. SSIM **(a)** and LPIPS **(b)** were calculated relative to the corresponding normal conditioning radiograph. Higher SSIM and lower LPIPS indicate greater similarity. Boxes show the interquartile range, horizontal lines indicate medians, × indicates means, and dots represent individual images. Mann–Whitney U test; *** indicates *p* < 0.001.

For image-conditioned generation, compared with the Gemini-image model, the GPT-image model generated synthetic radiographs with higher overall SSIM relative to the corresponding normal conditioning radiographs (mean: 0.681 [95% CI: 0.671–0.692] vs. 0.614 [95% CI: 0.605–0.623]; mean difference, 0.067; Mann–Whitney U test, *p* < 0.001) and lower overall LPIPS (mean: 0.226 [95% CI: 0.215–0.237] vs. 0.434 [95% CI: 0.422–0.445]; mean difference, −0.208; *p* < 0.001). This pattern was consistent across all disease categories and is shown in Figure 4.

For SSIM calculated relative to the corresponding normal conditioning radiographs, the GPT-image model showed higher values than the Gemini-image model for cardiomegaly (mean: 0.675 [95% CI: 0.654–0.697] vs. 0.616 [95% CI: 0.601–0.632]; *p* < 0.001), pneumothorax (mean: 0.683 [95% CI: 0.661–0.706] vs. 0.623 [95% CI: 0.604–0.643]; *p* < 0.001), pleural effusion (mean: 0.689 [95% CI: 0.670–0.709] vs. 0.617 [95% CI: 0.598–0.636]; *p* < 0.001), and pneumonia (mean: 0.678 [95% CI: 0.659–0.697] vs. 0.599 [95% CI: 0.581–0.617]; *p* < 0.001).

For LPIPS calculated relative to the corresponding normal conditioning radiographs, the GPT-image model showed lower values than the Gemini-image model for cardiomegaly (mean: 0.240 [95% CI: 0.217–0.264] vs. 0.436 [95% CI: 0.416–0.457]; *p* < 0.001), pneumothorax (mean: 0.226 [95% CI: 0.203–0.249] vs. 0.418 [95% CI: 0.393–0.443]; *p* < 0.001), pleural effusion (mean: 0.222 [95% CI: 0.200–0.243] vs. 0.431 [95% CI: 0.406–0.456]; *p* < 0.001), and pneumonia (mean: 0.217 [95% CI: 0.196–0.238] vs. 0.450 [95% CI: 0.427–0.472]; *p* < 0.001).

For FID, the GPT-image model showed lower values than the Gemini-image model under both generation strategies, indicating closer distributional similarity to real disease-positive radiographs. This pattern was consistent across disease categories and is shown in Table 3. As a reference baseline, split-half FID between real disease-positive radiograph subsets (160 vs. 160 radiographs) yielded a bootstrap mean of 43.40 (95% CI: 38.20–48.77) across 1,000 resamples.

**Table 3:** FID results by model, generation mode, and disease category.

Model	Generation mode	Cardiomegaly	Pneumothorax	Pleural effusion	Pneumonia	Overall
GPT	Text-only	151.85	149.77	152.18	152.95	118.24
	Image-conditioned	94.70	85.42	90.32	99.78	53.40
Gemini	Text-only	174.75	158.74	168.40	177.92	143.54
	Image-conditioned	154.02	113.03	135.94	142.05	104.76

Lower FID indicates greater similarity to real disease-positive radiographs. Overall FID was calculated using pooled images across all disease categories. FID, Fréchet Inception Distance.

### 3.5 Paired evaluation of previously undetected image-conditioned synthetic radiographs

In the paired evaluation of previously undetected image-conditioned synthetic chest radiographs, the overall AI detection rate was 79.1% (707/894; 95% CI: 76.3–81.6%) for the GPT-image model and 84.4% (672/796; 95% CI: 81.7–86.8%) for the Gemini-image model, corresponding to an absolute difference of 5.3% (two-proportion z test, *p* = 0.005). Across individual readers, AI detection rates ranged from 61.3 to 98.4% for the GPT-image model and from 69.0 to 97.1% for the Gemini-image model; the between-model difference was significant only for reader 4, with an absolute difference of 17.2% (Table 4).

**Table 4:** AI detection accuracy in the paired evaluation of real radiographs and previously undetected image-conditioned synthetic radiographs.

Reader	AI detection rate (GPT)	95% CI	AI detection rate (Gemini)	95% CI	*P-*value
Rad.1	187/190 (98.4%)	95.5–99.5	167/172 (97.1%)	93.4–98.8	0.391
Rad.2	208/225 (92.4%)	88.2–95.2	203/222 (91.4%)	87.0–94.5	0.697
Rad.3	125/174 (71.8%)	64.7–78.0	98/142 (69.0%)	61.0–76.0	0.584
Rad.4	187/305 (61.3%)	55.7–66.6	204/260 (78.5%)	73.1–83.0	< 0.001
Overall	707/894 (79.1%)	76.3–81.6	672/796 (84.4%)	81.7–86.8	0.005

Data are numbers of correctly identified AI-generated images, with percentages in parentheses. Confidence intervals were calculated with the Wilson method. *p-*values were calculated with the two-proportion z test.

## 4 Discussion

This study shows that conditioning on a normal chest radiograph substantially improves the perceived visual authenticity of finding-guided AI-generated chest radiographs. Compared with text-only generation, image-conditioned generation significantly reduced radiologists’ AI detection rates across both tested models and all four target abnormalities and produced lower FID values, indicating closer distributional similarity in feature space rather than clinical validity. The GPT-image model was less frequently identified as AI-generated than the Gemini-image model and showed higher image-level SSIM and lower image-level LPIPS relative to the conditioning radiographs, suggesting closer preservation of reference-image structure and perceptual appearance. These metrics reflect image-level similarity, not independent validation of disease correctness, anatomical plausibility, or pathological realism. Pneumothorax remained the most frequently detected target abnormality, indicating that fine pleural lines and subtle regional lung texture changes remain challenging for current generative models. In paired evaluation, most previously undetected image-conditioned images were identified as AI-generated, suggesting that subtle synthetic artifacts may become more apparent when a real comparator is available. The inter-reader agreement analysis further supports our main findings. Inter-reader agreement was low for text-only generation and even lower for image-conditioned generation, indicating that provenance assessment was reader-dependent.

The improved realism of image-conditioned generation likely reflects the structural and stylistic constraints provided by the real reference image (21). The credibility of a chest radiograph depends not only on the displayed abnormality but also on the consistency of normal anatomy, projection, grayscale distribution, and noise patterns. Text-only generation requires the model to synthesize both the radiographic background and the disease finding, increasing the likelihood of implausible anatomy, overly smooth texture, or discordant grayscale appearance (22, 23). In contrast, image-conditioned generation resembles disease editing of a normal radiograph, preserving patient-like anatomy and background texture while adding the specified target finding. Therefore, evaluations of synthetic medical images should distinguish text-only *de novo* synthesis from real-image-conditioned finding-guided editing, because these workflows have different implications for healthcare governance, medical education, dataset construction, and research integrity (24, 25).

The differences between the two models indicate that general-purpose GenAI systems can vary substantially in artifact patterns and medical image realism. Because commercial models are frequently updated and their training data and architectures are not fully transparent, performance should not be regarded as a stable property of a model name alone (26, 27). Models used for synthetic medical image generation or image-conditioned editing should be assessed using expert reader studies, finding-stratified analyses, and image-level quantitative metrics rather than selected examples or generic image-quality scores (28, 29). In this study, these metrics complemented the reader study and were not used to validate disease correctness. These assessments should be interpreted as evaluations of detectability and perceived image authenticity rather than as independent validation of disease severity, anatomical extent, or diagnostic correctness.

Although image-conditioned synthetic radiographs were difficult to identify when reviewed as isolated randomized images, side-by-side comparison improved detection, with rates of 79.1% for the GPT-image model and 84.4% for the Gemini-image model. This suggests that synthetic-image limitations may be overlooked when no paired real-image comparator is available. Paired real–synthetic comparison may therefore be useful for medical imaging education, synthetic-image quality assessment, and reader training focused on subtle inconsistencies in texture, noise, edges, and lesion morphology (30, 31). However, this setting differs from routine healthcare workflows, in which a matched real comparator is usually unavailable and image provenance is often assumed rather than actively verified. The paired setting therefore served as an artifact-awareness assessment, not as a test of clinical diagnostic accuracy.

Although formal cue-level error analysis was not prospectively collected, the detection patterns suggest several possible sources of synthetic-image recognition. Pneumothorax showed the highest AI-detection rate, possibly reflecting difficulty in generating convincing pleural lines and adjacent lung texture. More generally, radiologists may have relied on subtle inconsistencies in rib or clavicular contours, mediastinal or hilar borders, lung texture, image noise, edge sharpness, or lesion morphology. These observations should be interpreted as qualitative considerations rather than a formal per-image error analysis.

Synthetic chest radiographs may have constructive applications in healthcare, education, and research, including rare-case supplementation, finding-based teaching, dataset balancing, and simulation-based evaluation. Prior work suggests that synthetic medical images may improve dataset diversity and downstream model performance (32–34). However, these benefits require explicit labeling, expert review, and strict separation of synthetic and real datasets. If unlabeled synthetic radiographs enter teaching files, validation datasets, research repositories, or clinical documentation, they may introduce unrealistic disease patterns, model-specific artifacts, learning bias, dataset contamination, or overestimation of algorithmic performance (35).

The present findings also highlight a broader medical and ethical risk: image-conditioned generation may allow a user to transform a personal normal chest radiograph into a plausible disease-positive image. Such capability could be misused to fabricate evidence of illness for inappropriate medical claims, insurance fraud, occupational or disability certification, or other non-clinical advantages. Although this study did not evaluate fraudulent use directly, the low detection rate in isolated review indicates that visually plausible synthetic radiographs may challenge current verification practices if image provenance is not documented. This risk extends beyond radiology and is relevant to healthcare administration, insurance medicine, clinical research governance, and medical documentation systems.

These findings support a shift from human visual discrimination alone toward verifiable image provenance and controlled GenAI integration. As multimodal generative models lower the technical barrier to creating realistic medical images, human visual inspection alone is unlikely to be an adequate safeguard for image provenance. Healthcare and research workflows should incorporate provenance mechanisms such as DICOM metadata auditing, invisible watermarking, digital signatures, hash verification, image transfer logs, and dataset provenance records (36–38). The objective should be to make the origin, modification history, synthetic status, and intended use of each medical image traceable, especially when synthetic images are used for education, research, documentation, or dataset augmentation. Such measures may enable beneficial uses of GenAI while reducing the risk of unlabeled, inappropriate, or fraudulent integration into healthcare environments.

The enriched prevalence of synthetic images should be considered when interpreting our findings. Synthetic radiographs comprised 80% of the reader-study set, which facilitated comparisons across models and generation modes but does not reflect routine clinical practice. This design may have influenced reader vigilance and decision thresholds. Therefore, our results should be interpreted as relative detectability under controlled enriched conditions rather than real-world screening performance; in low-prevalence settings, the positive predictive value of a “synthetic” judgment would likely be lower.

This study has limitations. First, it was conducted at a single center with limited case and equipment diversity; multicenter validation is needed. Second, only four common chest radiographic abnormalities were included, and the findings may not generalize to other diseases or imaging modalities. Third, images were exported, cropped, and standardized to a uniform aspect ratio, which differs from native clinical DICOM viewing. Fourth, we assessed AI-origin detectability, reader-perceived visual authenticity, and visual similarity metrics but did not independently validate diagnostic correctness, disease severity consistency, anatomical extent, downstream educational value, or real-world misuse scenarios. In addition, the study did not prospectively record the specific visual cues underlying each reader judgment, so formal cue-frequency error analysis could not be performed. The radiologist reader study was label-informed and provenance-focused; it should not be interpreted as a *de novo* diagnostic task. Finally, commercial generative models evolve rapidly, and these results reflect only the tested model versions, API settings, prompt structure, preprocessing steps, and generation workflow.

In summary, image-conditioned generation substantially increased the reader-perceived visual authenticity of finding-guided AI-generated diseased chest radiographs, and the GPT-image model generated images that were less readily identified as AI-generated and showed greater similarity to the conditioning radiographs than those from the Gemini-image model. Although side-by-side comparison improved detection, the low detection rate during isolated review indicates that realistic synthetic radiographs may be difficult to recognize without explicit provenance information. Synthetic chest radiographs have potential value for medical education, dataset development, and healthcare research, but their use should occur within frameworks requiring transparent labeling, provenance tracking, expert review, and controlled integration into clinical, administrative, and research workflows.

## 5 Conclusion

In summary, image-conditioned generation substantially reduced the detectability of AI-generated diseased chest radiographs and improved apparent visual realism. Under the tested API settings, gpt-image-2 was less frequently identified as AI-generated than gemini-3-pro-image-preview and showed greater structural and perceptual similarity to the conditioning radiographs. Although side-by-side comparison improved detection, synthetic radiographs were often difficult to recognize in isolation. These findings highlight the need for transparent labeling, provenance tracking, expert review, and controlled use of AI-generated medical images in healthcare, education, and research.

## Data Availability

The original contributions presented in the study are included in the article/Supplementary material, further inquiries can be directed to the corresponding author.
