# Supplementary material for: Detectability and healthcare implications of generative AI–synthesized chest radiographs: a blinded radiologist reader study
Source: Front Med (Lausanne). 2026 Jul 15;13:1901949. doi: 10.3389/fmed.2026.1901949 (PMC13414275; doi:10.3389/fmed.2026.1901949)
Supplement: Supplementary file 1 [file supplementary_file_1.docx]

Supplementary Material

**Appendix S1.** Image preprocessing details

All source chest radiographs were exported from the PACS system at the highest available image quality. Each image was then center-cropped to a 1:1 aspect ratio while preserving the short side of the original image. Because sufficient peripheral background was present in all included cases, this cropping did not truncate the thoracic structures or the target pathology. The final square images retained high spatial resolution, with side lengths ranging approximately from 2,000 to 4,000 pixels depending on the original acquisition size (97.9% of images had a side length of no more than 3,000 pixels). The 1:1 aspect ratio was used to ensure compatibility with the output formats of the gpt-image-2 and gemini-3-pro-image-preview models and to facilitate standardized image presentation during the blinded reader study. This preprocessing was performed to standardize image inputs.

**Appendix S2.** Fréchet Inception Distance pipeline

Fréchet Inception Distance (FID) was calculated to assess the distribution-level similarity between synthetic disease-positive chest radiographs and the corresponding real disease-positive chest radiographs. FID was computed separately for each synthetic image set defined by generation model and generation strategy, and was additionally calculated within each disease category. Overall FID was calculated after pooling images across the four disease categories.

FID was calculated using the TorchMetrics implementation with the standard Inception-v3 feature extractor and 2048-dimensional feature representations. The Inception network used for FID was the conventional ImageNet-pretrained Inception-v3 model used in standard FID calculation. No chest-radiograph-specific pretraining or fine-tuning was applied to the Inception network.

All images were processed as three-channel RGB images before FID calculation. Although the chest radiographs were visually grayscale, the analyzed image files were stored in RGB mode. Channel-equality checks confirmed that the three channels contained identical information, with R = G and G = B. Therefore, no separate grayscale-to-RGB replication step was required. RGB conversion was used only to ensure a consistent three-channel input format for the Inception feature extractor.

Before FID calculation, both real and synthetic images were resized to 2048 × 2048 pixels using bilinear interpolation. Images were then provided to the FID pipeline as unsigned 8-bit image tensors with pixel values in the range 0–255. The TorchMetrics FID setting normalize=False was used. No additional intensity normalization, histogram equalization, min–max scaling, windowing, or ImageNet mean–standard deviation normalization was applied before FID calculation.

For each FID comparison, the real disease-positive radiographs constituted the reference distribution, and the corresponding synthetic disease-positive radiographs constituted the generated distribution. Text-only synthetic radiographs and image-conditioned synthetic radiographs were evaluated separately. Disease-specific FID values were calculated using only images from the corresponding disease category, whereas overall FID values were calculated using the pooled image sets across cardiomegaly, pneumothorax, pleural effusion, and pneumonia. FID was used only as a distribution-level image-similarity metric and was not interpreted as evidence of diagnostic correctness, disease severity accuracy, or clinical validity.

**Table S1.** Standardized prompt and API calling parameters for gpt-image-2

| **Item** | **Content** |
| --- | --- |
| **Parameter** | Model = gpt-image-2; n = 1; aspect ratio = 1:1; size = 2048 x 2048; output format = PNG; text-only generation: OpenAI Images API (/v1/images/generations); image-conditioned generation: OpenAI Images API (/v1/images/edits); generation dates: May 12-17, 2026. |
| **Prompt for text-only generation** | Generate a realistic frontal chest radiograph of an adult patient. Patient information: sex [sex], age [age] years. The radiograph should show the following imaging findings: [imaging findings]. The disease label is [disease label]. The image should resemble a real clinical chest X-ray, with appropriate grayscale contrast, projection, thoracic anatomy, lung texture, mediastinal contour, and radiographic noise. Do not add labels, arrows, annotations, text, watermarks, borders, or non-radiographic elements. |
| **Prompt for image-conditioned generation** | Using the provided normal frontal chest radiograph as the anatomical and stylistic reference, generate a realistic frontal chest radiograph of the same adult patient appearance with the following disease findings added. Patient information: sex [sex], age [age] years. Imaging findings to be represented: [imaging findings]. The disease label is [disease label]. Preserve the overall chest radiograph style, projection, anatomy, image texture, and grayscale appearance of the input radiograph as much as possible, while modifying the image to show the specified disease findings. Do not add labels, arrows, annotations, text, watermarks, borders, or non-radiographic elements. |
| **Note** | The raw prompts were input in Chinese. For each diseased case, patient age, sex, disease label, and imaging findings were extracted from the corresponding real radiology report and inserted into a standardized prompt template. Four disease labels were included: cardiomegaly, pneumothorax, pleural effusion, and pneumonia. For text-only generation, only the standardized text prompt was provided to the model. For image-conditioned generation, the same prompt was provided together with the corresponding age- and sex-matched normal frontal chest radiograph. All source images were center-cropped to a 1:1 aspect ratio before generation, and all synthetic images were saved as PNG files with a target size of 2048 x 2048 pixels. |

**Table S2.** Standardized prompt and API calling parameters for gemini-3-pro-image-preview

| **Item** | **Content** |
| --- | --- |
| **Parameter** | Model = gemini-3-pro-image-preview; aspect ratio = 1:1; image size = 2K; output format = PNG; text-only generation: Gemini API v1beta endpoint (/v1beta/models/gemini-3-pro-image-preview:generateContent); image-conditioned generation: Gemini API v1beta endpoint (/v1beta/models/gemini-3-pro-image-preview:generateContent); generation dates: May 12-17, 2026. |
| **Prompt for text-only generation** | Generate a realistic frontal chest radiograph of an adult patient. Patient information: sex [sex], age [age] years. The radiograph should show the following imaging findings: [imaging findings]. The disease label is [disease label]. The image should resemble a real clinical chest X-ray, with appropriate grayscale contrast, projection, thoracic anatomy, lung texture, mediastinal contour, and radiographic noise. Do not add labels, arrows, annotations, text, watermarks, borders, or non-radiographic elements. |
| **Prompt for image-conditioned generation** | Using the provided normal frontal chest radiograph as the anatomical and stylistic reference, generate a realistic frontal chest radiograph of the same adult patient appearance with the following disease findings added. Patient information: sex [sex], age [age] years. Imaging findings to be represented: [imaging findings]. The disease label is [disease label]. Preserve the overall chest radiograph style, projection, anatomy, image texture, and grayscale appearance of the input radiograph as much as possible, while modifying the image to show the specified disease findings. Do not add labels, arrows, annotations, text, watermarks, borders, or non-radiographic elements. |
| **Note** | The raw prompts were input in Chinese. For each diseased case, patient age, sex, disease label, and imaging findings were extracted from the corresponding real radiology report and inserted into a standardized prompt template. Four disease labels were included: cardiomegaly, pneumothorax, pleural effusion, and pneumonia. For text-only generation, only the standardized text prompt was provided to the model. For image-conditioned generation, the same prompt was provided together with the corresponding age- and sex-matched normal frontal chest radiograph. All source images were center-cropped to a 1:1 aspect ratio before generation, and all synthetic images were saved as PNG files. The image size parameter was set to 2K. |

**Table S3.** False-positive AI detection rates for real diseased chest radiographs in the single-image reader study

| **Reader** |  | **No. of evaluations** |  | **Incorrectly judged as AI-generated, n (%)** |
| --- | --- | --- | --- | --- |
| Overall |  | 1280 |  | 97 (7.6%) |
| Radiologist 1 |  | 320 |  | 20 (6.3%) |
| Radiologist 2 |  | 320 |  | 16 (5.0%) |
| Radiologist 3 |  | 320 |  | 24 (7.5%) |
| Radiologist 4 |  | 320 |  | 37 (11.6%) |


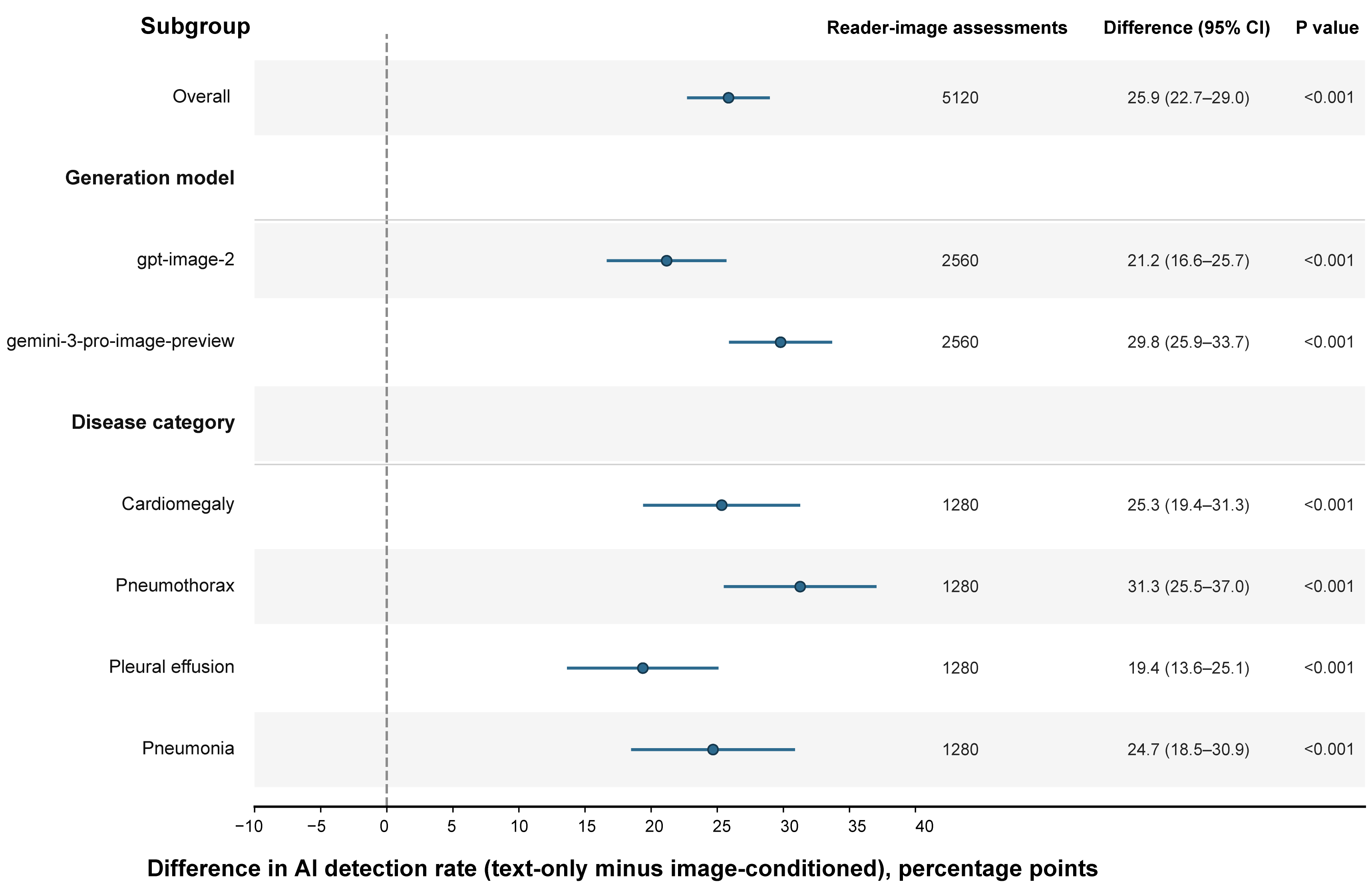


**Figure S1.** Difference in AI detection rates between text-only and image-conditioned synthetic chest radiographs. Dots show GLMM-adjusted marginal absolute differences in AI detection rate, calculated as text-only minus image-conditioned generation and expressed in percentage points; error bars show 95% confidence intervals. Positive values indicate lower AI detection rates for image-conditioned generation. The GLMM used a binomial distribution with logit link and included generation mode, generation model, disease label, age group, sex, and the generation mode × generation model × disease label interaction as fixed effects, with random intercepts for reader and source case. Marginal contrasts were estimated on the response scale using estimated marginal means. Reader-image assessments indicate the number of paired text-only versus image-conditioned evaluations contributing to each contrast.
